# Hilbert space multidimensional modelling of continuous measurements

**DOI:** 10.1098/rsta.2019.0142

**Published:** 2019-09-16

**Authors:** J. R. Busemeyer, Z. Wang

**Affiliations:** 1 Indiana University, Bloomington, IN, USA; 2 The Ohio State University, Columbus, OH, USA

**Keywords:** context effects, quantum probability, Kolmogorov probability

## Abstract

Data fusion problems arise when a researcher needs to analyse results obtained by measuring empirical variables under different measurement contexts. A context is defined by a subset of variables taken from a complete set of variables under investigation. Multiple contexts can be formed from different subsets, which produce a separate distribution of measurements associated with each context. A context effect occurs when the distributions produced by the different contexts cannot be reproduced by marginalizing over a complete joint distribution formed by all the variables. We propose a Hilbert space multidimensional theory that uses a state vector and measurement operators to account for multiple distributions produced by different contexts.

This article is part of the theme issue ‘Contextuality and probability in quantum mechanics and beyond’.

## 1. Introduction

A measurement context refers to a set of empirical variables that are measured on the same occasion [1]. Different contexts are formed by measuring different overlapping subsets of variables. A context effect occurs when measurements are affected by the context in which they appear (this is defined more clearly in §2). This can happen when the reaction to the measurement of one variable changes depending on which other variables are being measured in the same context.

To give an empirical example in psychology, consider the following study reported in [2] that investigated the effectiveness of persuasive messages. Participants were asked to judge four attributes: how persuasive, believable, informative and likeable the messages were perceived to be. Each attribute is an empirical variable. A context was formed by asking about a pair of attributes regarding the same message (e.g. Is the message likeable and persuasive? Answer yes or no separately to each of the two attributes).

Each pair of attributes produced a 2 × 2 joint frequency table (frequency of (*yy*, *yn*, *ny*, *nn*) pairs of answers). Six different contexts were produced by asking for evaluations of six pairs of attributes (e.g. Is the message likeable and persuasive? Is the message believable and persuasive?). The six contexts produced six different 2 × 2 joint frequency tables. We proposed a ‘context-free’ model that assumed four binary random variables, *P*, *B*, *I*, *L* producing a four-way joint distribution containing 16 joint probabilities (e.g. *p*(*P* = *y*, *B* = *y*, *I* = *n*, *L* = *n*)). We statistically tested whether this context-free model could reproduce the 6 two-way tables (e.g. using the marginal probabilities *p*(*P* = *i*, *B* = *j*), *i* = (*y*, *n*), *j* = (*y*, *n*)) to predict the two-way, persuasive and believable, pair of attributes table). Based on a log-likelihood ratio *χ*^2^ test, we found statistically significant deviations from the context-free model, which indicated that the interpretation of a message attribute (e.g. persuasive) changed depending on the other attribute with which it was paired (likeable versus believable) [2].

To give another example, consider a study reported in [3] that investigated evaluations of avatar agents. Participants observed pictures of female avatars and made binary judgements about attractiveness, intelligence, sociability and honesty of each avatar. For each presentation of an avatar, the participant was asked to judge a pair of attributes (e.g. judge attractiveness and intelligence of this avatar). Judging all six pairs of attributes produced a total of six contexts. Once again we proposed a context-free model that assumed four binary random variables, *A*, *I*, *S*, *H*, producing a four-way joint distribution containing 16 joint probabilities (e.g. *p*(*A* = *y*, *I* = *n*, *S* = *y*, *H* = *n*)). We statistically tested whether this context-free model could reproduce the six two-way tables (e.g. using the marginal probabilities *p*(*A* = *j*, *H* = *k*), *j* = (*y*, *n*), *k* = (*y*, *n*)) to predict the two-way, attractive and honest, pair of attributes table). Again, we found statistically significant deviations from the context-free model, which indicated that the interpretation of an avatar attribute (e.g. intelligent) changed depending on the other attribute with which it was paired (attractive versus honest).

As a third example, we [4] examined context effects obtained from rating effectiveness of a message from a ‘self’ versus ‘other’ perspective in two different question orders. The self/other perspectives define two empirical variables, and the context was manipulated by the order of perspective presentation. This study used a nine-point rating scale, which produced two 9 × 9 joint frequency distributions, one distribution for each order. The difference between the two distributions was statistically significant, indicating a context effect produced by a question order effect.

To account for these results, we developed a theory, called the Hilbert space multidimensional (HSM) theory [2–4]. HSM theory attempts to provide a single coherent representation of all the empirical variables measured in various contexts and makes predictions for new contexts. The theory uses a single state vector to represent a person's belief state, and a measurement operator to represent each variable. The predictive accuracy of the models developed from the HSM theory was compared to Bayes net and Markov models using the same number of parameters. The parameters were estimated using maximum-likelihood methods, and the HSM models were found to produce better predictions than the Bayes net and Markov models.

So far, HSM theory has been developed only for discrete and finite empirical variables [2–4]. Many variables of interest to social and behavioural scientists are approximately continuously distributed. Here, we extend the HSM theory to continuous variables.

## 2. The context-free probability model

Consider the following data fusion type of problem that arises from the use of what we call ‘contextual’ measurements. Suppose we have three different sensors used to measure the psycho-physiological state of a person, denoted *X*, *Y*, *Z*. Each sensor is called an empirical variable because it is simply a measuring device. A sensor produces measurement outcomes, which are recorded as real numbers (approximately at least). The measurement outcome of a sensor defines an empirical event such as ‘the sensor was observed to produce a value less than *x*’. We may have to apply the sensors one at a time. Furthermore, the person may react to sensors, and, therefore, be sensitive to the sequence of measurements.

The collection of empirical measurement outcomes that can be produced by the sensors, applied under some measurement context, produces a sample space of outcomes, upon which we can define random variables. Following [1], the notation, *X*^context^, *Y*^context^, *Z*^context^, refers to three different random variables measured in a *context*. For example, [*x*] denotes a context in which a single sensor *X* is measured alone, which produces the random variable *X*^[*x*]^; the pair [*x*, *y*] denotes a context in which two sensors are measured sequentially in the order of sensor *X* first, sensor *Y* second, which produces two random variables *X*^[*x*,*y*]^, *Y*^[*x*,*y*]^; the triple [*x*, *y*, *z*] denotes a context in which three sensors were measured sequentially in the order indicated by *X* first, *Y* second, *Z* third, which produces three random variables *X*^[*x*,*y*,*z*]^, *Y*^[*x*,*y*,*z*]^, *Z*^[*x*,*y*,*z*]^. If the sequence does not matter, e.g. *X*^[*x*,*y*]^ = *X*^[*y*,*x*]^, then we can describe the context by a set {*x*, *y*} rather than a tuple [*x*, *y*] and use a random variable defined as *X*^{*x*,*y*}^.

If we measure sensor *X* alone, then we obtain an empirical estimate of the distribution *F*_*X*_(*x*) = *Pr*[*X*^[*x*]^ ≤ *x*]; likewise, if we measure sensors *Y* alone or *Z* alone, then we obtain estimates of distributions *F*_*Y*_(*y*) and *F*_*Z*_(*z*), respectively. When we measure sensor *X* and then sensor *Y*, we obtain an estimate of a two-way distribution *F*_*XY*_(*x*, *y*) = *Pr*[*X*^[*x*,*y*]^ ≤ *x*, *Y*^[*x*,*y*]^ ≤ *y*], which could differ from the distribution *F*_*YX*_(*y*, *x*) = *Pr*[*Y*^[*y*,*x*]^ ≤ *y*, *X*^[*y*,*x*]^ ≤ *x*] obtained from the opposite order. Likewise, when we measure sensors *Y* and then *Z*, we obtain an estimate of a two-way distribution *F*_*YZ*_(*y*, *z*), and the measurement of *X* and then *Z* produces *F*_*XZ*_(*x*, *z*). Finally, we could measure all three sensors in the sequence [*x*, *y*, *z*], to produce an estimate of a distribution *F*_*XYZ*_(*x*, *y*, *z*) = *Pr*[*X*^[*x*,*y*,*z*]^ ≤ *x*, *Y*^[*x*,*y*,*z*]^ ≤ *y*, *Z*^[*x*,*y*,*z*]^ ≤ *z*]. Considering only the distributions explicitly mentioned above (which does not include all possible distributions) the three one-way, four two-way and one three-way empirical distributions mentioned above involve a total of 14 different random variables to describe the collection of context-dependent distributions. We would like to construct an empirically testable, more coherent, and more parsimonious probability model that could accurately predict all of these separate one-, two- and three-way context-dependent distributions.

It is common (see, e.g. Bayes net models, [5]) to try to reproduce a collection of probability distributions, such as that obtained from our example with three sensors, by a ‘context-free’ model, defined as follows. We postulate a single joint distribution function defined over three context-free random variables, *X*^*f*^, *Y*^*f*^, *Z*^*f*^ (where *f* stands for ‘free’), one corresponding to each sensor, with joint distribution *F*(*x*, *y*, *x*) = *Pr*[*X*^*f*^ ≤ *x*∩*Y*^*f*^ ≤ *y*∩*Z*^*f*^ ≤ *z*]. First, this three-way, ‘context-free’ distribution would need to reproduce the sequential *F*_*XYZ*_(*x*, *y*, *z*) distribution. Additionally, this three-way joint distribution would need to reproduce the two-way distributions by marginalization, e.g. *F*_*XY*_(*x*, *y*) = *F*(*x*, *y*, ∞), *F*_*YZ*_(*y*, *z*) = *F*(∞, *y*, *z*) and *F*_*XZ*_(*x*, *z*) = *F*(*x*, ∞, *z*). Similarly, the single random variable distributions would also be obtained by marginalization, e.g. *F*_*X*_(*x*) = *F*(*x*, ∞, ∞).

The ‘context-free’ model, however, may fail to reproduce the collection of one, two and three-way empirical distributions. This can occur because such a construction requires the different distributions to satisfy too many consistency constraints. We define context effects by these violations of the consistency constraints imposed by the ‘context-free’ model. Statistically, significant deviations from the ‘context-free’ model indicate the presence of context effects. There are different kinds of context effects.

First of all, the ‘context-free’ model requires the distribution of the random variables *X*^*f*^, *Y*^*f*^, *Z*^*f*^ to be insensitive to the sequence of measurement, e.g. it predicts that *F*_*XY*_(*x*, *y*) = *F*_*YX*_(*y*, *x*) = *F*(*x*, *y*, ∞).

Second, even if the sequence does not empirically matter, the context-free model may fail because of violations of marginal invariance, e.g. *F*_*Y*_(*y*)≠*F*_*XY*_(∞, *y*), or perhaps *F*_*XY*_(∞, *y*) ≠*F*_*YZ*_(*y*, ∞). This could occur if the meaning or interpretation of one sensor changes depending on the context created by the other sensor.

Finally, even if there are no sequence effects, and marginal invariance holds, violations of correlation constraints (Bell type of inequalities) required by the ‘context-free’ model can occur. For example, see Theorem 2 in [6], and for a more general summary of constraints see [1].

## 3. Comparison of classical, quantum and Hilbert space multidimensional theories

### (a) Comparison of classical and quantum probability

HSM theory is based on quantum probability, and so it is useful to review the fundamental axioms of quantum probability and compare them with classical probability axioms. The purpose of a probability theory is to assign probabilities to events that we observe in the world. Classic (Kolmogorov) probability theory provides one way to do this, and quantum (von Neumann) probability theory provides a second way. Although classic theory has been more widely applied than quantum theory outside of physics, both theories are based on general axioms and thus they are both potentially applicable to a wide range of problems. Both theories are applicable to finite, countably infinite, or uncountably infinite spaces depending on the application. In our previous articles, we treated finite, discrete empirical variables [2,3]. However, this application also works with continuously distributed variables.

Classic probability theory was designed with classical events in mind (e.g. path of a bullet). Although classic probability theory was originally developed by Pascal, Bernoulli, Huygens, Laplace, Gauss and many others across the span of 300 years, an axiomatic development was first provided by Kolmogorov in 1933 [7].

The principal idea of Kolmogorov theory is that events are formally represented as subsets of a sample space *Ω*. The collection of events includes the empty set ⊘ and it is assumed to be closed under complementation and countable union to form a *σ* − *algebra*, denoted Σ, of events. A probability measure is a non-negative real-valued function *s* on Σ satisfying *s*(*Ω*) = 1 and for a countable number of indices, if *A*_*i*_∈Σ and *A*_*i*_∩*A*_*j*_ = ⊘ for *i*≠*j* then $s(\cup A_{i})=\sum s(A_{i})$. When we take measurements of a probabilistic system (like a person), the probability function *s* is also called the *state* of the system.

Quantum probability theory was designed with quantum events in mind (e.g. the ‘path’ of an electron). Although quantum probability theory was originally developed by physicists such as Heisenberg, Schrödinger, Born, Dirac and others early in the twentieth century, an axiomatic development was first provided by von Neumann in 1932 [8].

The principal idea of quantum theory is that events are formally represented as subspaces of a Hilbert space H. A complex Hilbert space is a complete inner product vector space defined on a complex field. Here, the space consists of the continuous square integrable complex-valued functions (e.g. *ψ*, *ϕ*) defined on a bounded interval [*l*, *u*], and the inner product is defined as

3.1 $$\langle \psi \;|\;\phi \rangle =\int_{l}^{u}\psi {\left(x\right)}^{*}\cdot \phi (x)\cdot dx,$$

(x* is the conjugate of *x*). Each subspace L⊂H corresponds to a projector operator *P*_*L*_, which satisfies the properties *P*_*L*_ = *P*_*L*_ · *P*_*L*_ = *P*^†^_*L*_. (*P*^†^ is the Hermitian adjoint of the linear operator *P*.) The collection of all projectors, denoted *Φ*, forms a partial Boolean algebra of events. The *state* of a quantum system is defined as a ray spanned by a unit length vector ψ∈H. The state can also be described as a one-dimensional projector denoted *ρ* = *ψ* · *ψ*^†^, where *ψ*^†^ is the vector in the dual space of H corresponding to *ψ*.^1^ The probability of an event L⊂H is derived from the state *ρ* using the following quantum algorithm:

3.2 $$s(L)=\mathrm{tr}[P_{L}\rho P_{L}^{†}].$$

(Although *P*^†^_*L*_ = *P*_*L*_ for projectors, later it will help to retain the adjoint.)

The quantum algorithm satisfies the following requirements for a probability measure. First, like classical probabilities, quantum probabilities are non-negative because *P*_*L*_ is a positive operator and *s*(*L*) = tr[*P*_*L*_*ρP*^†^_*L*_]≥0. Second, similar to the classical probability requirement that the sample space is assigned a probability equal to one, note that the identity operator *I*, defined by *I* · *ψ* = *ψ*, projects onto H and so s(H)=tr[I⋅ρ⋅I]=1; also, like the additive property of classical theory, if *L*_*i*_ and *L*_*j*_ for *i*≠*j* are orthogonal so that *P*_*i*_ · *P*_*j*_ = **0**, then $s(\vee L_{i})=\mathrm{tr}[(\sum P_{i})\rho {\left(\sum P_{i}\right)}^{†}]=\sum \mathrm{tr}[P_{i}\rho P_{i}^{†}]=\sum s(L_{i})$, where *L*_*i*_∨*L*_*j*_ denotes the span of the two subspaces. The quantum algorithm is not an arbitrary choice; it turns out that for vector spaces with dimension greater than two, all probability measures for events defined as subspaces can be computed this way [9].

According to quantum theory, two events *L*_*A*_ and *L*_*B*_ are commutative if their commutator is zero, *P*_*A*_*P*_*B*_ − *P*_*B*_*P*_*A*_ = 0. The events *L*_*A*_ and *L*_*B*_ are non-commutative if their commutator is not zero, *P*_*A*_*P*_*B*_ − *P*_*B*_*P*_*A*_≠0. In quantum theory, compatible is the same as commutative, and incompatible is the same as non-commutative.

In Kolmogorov theory, a random variable, such as for example *X*^[context]^, is a real-valued measurable function defined on *Ω*. In quantum theory, each empirical variable, such as the sensor *X*, corresponds to an observable, **X**, which is a Hermitian operator, **X**^†^ = **X**. A bounded Hermitian operator always has a spectral decomposition, and here we assume it has a continuous spectrum, $X=\int_{x=l}^{x=u}x\cdot \;dP(x)$ where *x*∈[*l*, *u*] is a real spectral value and $P(a)=\int_{l}^{a}\;dP(x)$ for *l* ≤ *a* ≤ *u* is the projector for the empirical event ‘observing a sensor value from *X* that is less than or equal to *x*’ [10]. If two observables, **X**, **Y**, commute, then they are described as compatible, and otherwise they are incompatible.

The structure of the Hilbert space depends on the commutative relations among the pairs of observables. For a pair of commutative obserables, we follow quantum mechanics (see, e.g. [11], ch. 1) and assume that they can be decomposed into a common refinement of projectors $X=\int_{y=l}^{y=u}\int_{x=l}^{x=u}f(x,y)\cdot dP(x,y)$ and $Y=\int_{y=l}^{y=u}\int_{x=l}^{x=u}g(x,y)\cdot dP(x,y)$ with *f*(*x*, *y*) = *x*, *g*(*x*, *y*) = *y*, and *P*(*x*, *y*) = *P*_*X*_(*x*) · *P*_*Y*_(*y*). For a pair of non-commutative observables, we assume that they are unitarily equivalent :**Y** = *U* · **X** · *U*^†^. All unitary operators *U*_*X*_ satisfy the properties *U*_*X*_*U*^†^_*X*_ = *U*^†^_*X*_*U*_*X*_ = *I*. See [10, (p. 150)]), for the requirements of unitary equivalence. In practice, this requires us to scale all our sensory variables between [*l*, *u*].

Some well-known examples of observables in physics are the position and momentum observables. For a given position spatial coordinate, say the *x* position coordinate, both the position and momentum observables have a continuous spectrum. However, they are non-commutative, and so they are related by a unitary transformation, which happens to be the standard Fourier transformation. Two different spatial position coordinates, say the *x* and *y* coordinates, are compatible and both observables can be decomposed into a common family of projectors.

So far, we have simply summarized some basic principles from quantum probability theory. The next section identifies the contribution of HSM theory and how it steps outside the physical theories.

### (b) Contribution of multidimensional Hilbert space theory

The goal of HSM theory, as described in §§4–7, is to lay out the basic mathematical steps used to build HSM models for continuous empirical variables. HSM theory makes two new contributions outside of standard quantum probability theory described above. One is to provide a guide to applications of quantum theory outside of physics to social and behavioural sciences. Most scientists, whether behavioural, social or even physical, have little or no idea about how to apply quantum theory outside of physics. We built a general program for doing this, which started with discrete and finite empirical variables. We had reasonable success with discrete, finite variables, and this presentation extends the program to (approximately) continuous empirical variables. The second contribution is that HSM theory may encounter unitary operators that do not arise in physics (see, e.g. §6a), and so we need to describe the use of unitary transformations that do not appear in physics. This is needed to deal with new kinds of empirical variables appearing in the social and behavioural sciences. It is important to point out that the general framework is intended to be a guide for building specific models. The specific models built from these principles are then used to make predictions that are empirically testable. Section 8 describes successful implementations of these principles to psychology experiments using (approximately) continuous measures.

Outside of physics, we do not know which empirical variables commute and which do not. Empirically, we determine whether or not a pair of variables commutes by experimentally testing for sequential effects of measurement. If the measurement sequence affects the observed probability distribution, then we consider a model which assumes that the observables do not commute. Otherwise, we assume that they commute. Although we establish the commutative relation *post hoc*, an HSM model provides empirically testable predictions concerning the resulting probability distributions. For example, if all three observables are non-commutative, then once we formulate a specific parameterized model and determine the model parameters for predicting the empirical distributions for context [*x*, *y*] and [*y*, *z*], we can use these same parameters to predict the empirical distributions for contexts [*x*, *z*] and [*x*, *y*, *z*].

Our HSM program for constructing multiple probability distributions from a single state *ρ* using quantum theory proceeds as follows. In §4, we discuss how to compute the probability distributions depending on the commutative relations among the observables. In §5, we define the projectors *P*_*L*_ for each observable, which is done by constructing them from a unitary operator, *U*. In §6, we discuss a method for constructing unitary operators, and this is done by defining a self-adjoint Hermitian operator *H*. So, in §6, we also discuss methods for building a Hermitian operator. Finally, in §7, we propose a method for constructing the initial quantum state *ρ* used to generate all of the probability distributions from the quantum algorithm.

## 4. Step 1: Computing probability distributions

Probabilities in quantum theory are computed by first defining the possible measurement outcomes that can be produced by measuring an empirical variable, then assigning a projector to each unique measurement outcome, and finally applying these projectors to the state in the sequence determined by the context.

Continuing with our example, we are considering three sensors (empirical variables), *X*, *Y*, *Z*. If we measure sensor *X*, we can observe the empirical event that *X* is less than or equal to *x* (i.e. *X* ≤ *x*). If we measure *Y* or *Z* we can observe empirical events, *Y* ≤ *y* and *Z* ≤ *z*. Define *P*_*X*_(*x*) as the projector corresponding to the empirical event *X* ≤ *x*, define *P*_*Y*_(*y*) as the projector corresponding to the event *Y* ≤ *y*, and *P*_*Z*_(*z*) as the projector corresponding to the event *Z* ≤ *z*.

### (a) All three commutative pairs

Let us first examine the situation when the events of interest are all compatible (all the observables commute with each other). In physics, this would correspond to the situation, for example, when measurements of position are taken on each of the three different spatial dimensions. In this case, all three observables can be decomposed into the same refined family of projectors. The Hilbert space, in this case, is denoted $H^{3}$, because it has three pairs of commuting observables. The projectors *P*_*X*_(*x*), *P*_*Y*_(*y*) and *P*_*Z*_(*z*) for the three observables all operate in the space $H^{3}$. In this case, the quantum model predicts no sequence effects or context effects at all, and it is equivalent to the context-free classical model.

The product of two projectors, *P*_*X*∩*Y*_(*x*, *y*) = *P*_*X*_(*x*) · *P*_*Y*_(*y*) is also a projector when they commute. In the context {*x*, *y*}, this product is used to predict the joint probability

$$G(x,y)=\mathrm{tr}[P_{X\cap Y}(x,y)\cdot \rho \cdot P_{X\cap Y}{\left(x,y\right)}^{†}].$$

This model predicts *F*_*XY*_(*x*, *y*) = *F*_*YX*_(*y*, *x*) = *G*(*x*, *y*), that is, an insensitivity to order, just like the context-free classical model.

The product of three projectors *P*_*X*∩*Y* ∩*Z*_(*x*, *y*, *z*) = *P*_*Z*_(*z*) · *P*_*X*∩*Y*_(*x*, *y*) is also a projector when they all commute. In the context {*x*, *y*, *z*}, the joint probability of all three events equals

$$G(x,y,z)=\mathrm{tr}[P_{X\cap Y\cap Z}(x,y,z)\cdot \rho \cdot P_{X\cap Y\cap Z}{\left(x,y,z\right)}^{†}].$$

Once again this model predicts *F*_*XYZ*_(*x*, *y*, *z*) = *F*_*ZYX*_(*z*, *y*, *x*) = *G*(*x*, *y*, *z*), that is an insensitivity to sequence, just like the context-free classical probability model. This model can reproduce all of the two-way and single marginal distributions only if there are no context effects. If all the projectors commute, then there is no difference between the quantum and context-free model.

### (b) All three non-commutative pairs

Next, we examine the situation when all three pairs of observables do not commute. Their projectors are represented in different bases related by unitary transformations. In physics, this situation occurs when the spin of a particle is measured along three different angles. The Hilbert space is simpler in this case, and it will be denoted as H. The projectors *P*_*X*_(*x*), *P*_*Y*_(*y*) and *P*_*Z*_(*z*) all operate in space H.

Suppose we measure sensor *X*, and then sensor *Y*, and we wish to predict the empirical probability of the event that sensor *X* ≤ *x* and then sensor *Y* ≤ *y* occurs in context [*x*, *y*]. If we assume that these two projectors do not commute, then the product of the two projectors, *Q*_*X*,*Y*_(*x*, *y*) = *P*_*Y*_(*y*) · *P*_*X*_(*x*), is *not* a projector, so that the joint event does *not* exist. The predicted sequential probability equals

$$G_{XY}(x,y)=\mathrm{tr}[Q_{X,Y}(x,y)\cdot \rho \cdot Q_{X,Y}{\left(x,y\right)}^{†}].$$

Because of the cyclic property of the trace operator, tr[*AB*] = tr[*BA*], we can rewrite the above probability as

$$\mathrm{tr}[Q_{X,Y}(x,y)\cdot \rho \cdot Q_{X,Y}{\left(x,y\right)}^{†}]=\mathrm{tr}[Q_{X,Y}{\left(x,y\right)}^{†}Q_{X,Y}(x,y)\rho ]$$

and *Q*^†^_*X*,*Y*_*Q*_*X*,*Y*_ is a positive operator valued measurement collection (POVM). This is a more general type of measurement, and *Q*_*X*,*Y*_(*x*, *y*)^†^ · *Q*_*X*,*Y*_(*x*, *y*) is an element of this POVM.

The key contribution of quantum theory is that it accounts for path dependency by applying a sequence of non-commuting projectors. The probability of the event that sensor *Y* ≤ *y* and then sensor *X* ≤ *x* occurs in context [*y*, *x*] is determined by the opposite sequence of projections. Thus, if we define *Q*_*Y*,*X*_(*y*, *x*) = *P*_*X*_(*x*) · *P*_*Y*_(*y*), then

$$G_{YX}(y,x)=\mathrm{tr}[Q_{YX}(y,x)\cdot \rho \cdot Q_{YX}{\left(y,x\right)}^{†}].$$

Because the projectors do not commute, *Q*_*Y*,*X*_ does not equal *Q*_*X*,*Y*_. It remains an empirical question whether or not the quantum model can reproduce the distributions *F*_*XY*_(*x*, *y*) and *F*_*YX*_(*y*, *x*) using the predicted distributions *G*_*X*,*Y*_(*x*, *y*) and *G*_*Y*,*X*_(*y*, *x*).

The product of three projectors *Q*_*X*,*Y*,*Z*_(*x*, *y*, *z*) = *P*_*Z*_(*z*) · *Q*_*X*,*Y*_(*x*, *y*) is used to determine the probability of the sequence of three events produced by measuring sensors *X* then *Y* and then *Z* in context [*x*, *y*, *z*], as follows

$$G_{XYZ}(x,y,z)=\mathrm{tr}[Q_{X,Y,Z}(x,y,z)\cdot \rho \cdot Q_{X,Y,Z}{\left(x,y,z\right)}^{†}].$$

The measurement *Q*_*X*,*Y*,*Z*_ is again a type of POVM. Because the projectors do not commute, the operators of this measurement depend on the sequence which the three empirical variables are measured.

This model provides a strong *a priori* parameter free test. We can initially estimate the parameters used to construct *P*_*X*_(*x*), *P*_*Y*_(*y*), and *P*_*Z*_(*z*) from the fits of the empirical distributions *F*_*XY*_(*x*, *y*) and *F*_*YZ*_(*y*, *z*) to specific parameterized HSM models. Then we can use the same parameters to make *a priori* predictions for the empirical distribution *F*_*XZ*_(*x*, *z*), because the latter is obtained by the product of projectors *P*_*X*_(*x*) and *P*_*Z*_(*z*).

### (c) Two commutative pairs and one non-commutative pair

Now suppose that the observables **X** and **Z** commute, and also **Y** and **Z** commute, but **X** and **Y** do not commute. This is analogous to the physical situation in which we measure position and then momentum on the same axis, and also measure position on a different axis. The Hilbert space, in this case, will be denoted $H^{2}$, because it has two pairs of commuting observables. The projectors *P*_*X*_(*x*), *P*_*Y*_(*y*), *P*_*Z*_(*z*) all operate in the space $H^{2}$. In this case, the observables **X** and **Z** can be decomposed into the same family of projectors; similarly, the observables **Y** and **Z** can be decomposed into the same family of projectors. However, the observable **Y** is a unitary transformation of the observable **X**.

Suppose we are considering the two commuting observables, such as **X** and **Z**, and we are concerned with the probability that sensor *X* ≤ *x* and sensor *Z* ≤ *z* occur in the context {*x*, *z*}. The product of the two projectors, *P*_*X*∩*Z*_(*x*, *z*) = *P*_*X*_(*x*) · *P*_*Z*_(*z*) is also a projector so that the joint event exists, and the joint probability equals

$$G_{XZ}(x,z)=\mathrm{tr}[P_{X\cap Z}(x,z)\cdot \rho \cdot P_{X\cap Z}{\left(x,z\right)}^{†}].$$

The predicted probabilities for this model are order free *G*_*ZX*_(*z*, *x*) = *G*_*XZ*_(*x*, *z*), because the projectors commute. The same ideas apply to the other pair of commuting observables **Y**, **Z**.

Now consider the two non-commuting observables, **X** and **Y**, and we are concerned with the probability that sensor *X* ≤ *x* and then sensor *Y* ≤ *y* in context [*x*, *y*]. If we assume that these two projectors do not commute, then the product of the two projectors, *Q*_*X*,*Y*_(*x*, *y*) = *P*_*Y*_(*y*) · *P*_*X*_(*x*), is *not* a projector, so that the joint event does *not* exist, and the sequential probability equals

$$G_{XY}(x,y)=\mathrm{tr}[Q_{X,Y}(x,y)\cdot \rho \cdot Q_{X,Y}{\left(x,y\right)}^{†}].$$

The probability of a third event *Z* ≤ *z* in context [*x*, *y*, *z*] then equals

$$G_{XYZ}(x,y,z)=\mathrm{tr}[P_{Z}(z)Q_{X,Y}(x,y)\cdot \rho \cdot Q_{X,Y}{\left(x,y\right)}^{†}P_{Z}{\left(z\right)}^{†}].$$

Note that if all three are measured, then the order that we measure *Z* does not matter,

$$\begin{array}{rl} G_{X,Y,Z}(x,y,z) & =\mathrm{tr}[P_{Z}(z)Q_{X,Y}(x,y)\cdot \rho \cdot Q_{X,Y}{\left(x,y\right)}^{†}P_{Z}{\left(z\right)}^{†}]\\ & =\mathrm{tr}[Q_{X,Y}(x,y)P_{Z}(z)\cdot \rho \cdot P_{Z}{\left(z\right)}^{†}Q_{X,Y}{\left(x,y\right)}^{†}]\\ & =G_{Z,X,Y}(z,x,y), \end{array}$$

and only the order of *X* and *Y* matters.

This model can also provide a strong *a priori* empirically testable prediction. We can estimate the parameters needed to construct *P*_*X*_(*x*), *P*_*X*_(*x*) and *P*_*X*_(*x*) by fitting the empirical two-way distributions, and then use these same parameters to generate an *a priori* new prediction for the three-way distributions.

## 5. Step 2: Constructing non-commutative projectors

To apply quantum theory, we need an algorithm for constructing the projectors. This depends on the commutative relations among the projectors. Let us again consider three continuous empirical sensor variables, *X*, *Y*, *Z*.

### (a) All three non-commutative pairs

We will start with the assumption that the three sensor variables correspond to three observables, **X**, **Y**, **Z**, that are all pairwise non-commutative. We assume that the space H is the completion of the space of complex square integrable continuous functions ψ∈H on [*l*, *u*]⊂*R*. The function value of ***ψ*** at the real index value *w* has a coordinate *ψ*(*w*), which is the complex amplitude assigned to index *w*. We define a projector *P*(*a*) on an arbitrary real-valued interval [*l*, *a*], *l*≤*a* as follows:

$$P(a)\cdot \psi =\psi_{a}$$

and

$$\psi_{a}(w)=\left\{ \begin{array}{ll} \psi (w) & l\leq w\leq a\\ 0 & \mathrm{otherwise}. \end{array}\right.$$

The projector for the measurement outcomes for observable **X** on the interval [*l*, *x*] is then constructed from a unitary operator *U*_*X*_

5.1 $$P_{X}(x)=U_{X}P(x)U_{X}^{†}.$$

Note that to predict context effects, *U*_*X*_ is chosen so that *P*(*a*)*U*_*X*_ does equal *U*_*X*_*P*(*a*) for at least two of the three observables. Also note that *P*_*X*_(*x*) = *P*_*X*_(*x*)^†^ and *P*_*X*_(*x*)*P*_*X*_(*x*) = *P*_*X*_(*x*), which guarantees that it is a projector. Essentially, the unitary operator *U*_*X*_ defines the basis of the vector space used to define the events for sensor *X*. Likewise, the projectors for sensors *Y* and *Z* on an interval [*l*, *a*] are constructed from unitary operators *P*_*Y*_(*a*) = *U*_*Y*_*P*(*y*)*U*^†^_*Y*_, and *P*_*Z*_(*a*) = *U*_*Z*_*P*(*z*)*U*^†^_*Z*_. Note that if *U*_*Y*_ = *U*_*Z*_ then the two variables commute.

### (b) Two commutative pairs and one non-commutative pair

Above we assumed that all three pairs of observables are non-commutative. Now suppose that **X**, **Y**, do not commute, but **Z** commutes with both.

In this case, we assume that the Hilbert space $H^{2}$ completion of the space of complex square integrable continuous functions $\psi \in H^{2}$ on [*l*, *u*]^2^⊂*R*^2^ with complex coordinates *ψ*(*v*, *w*) defined on the pair of real value indices (*v*, *w*). We can define a projector *P*(*a*, *b*) for the space $H^{2}$ on the rectangle [*l*, *a*] × [*l*, *b*] as follows:

$$P(a,b)\cdot \psi =\psi_{a,b}$$

and

$$\psi_{a,b}(v,w)=\left\{ \begin{array}{ll} \psi (v,w) & l\leq v\leq a,l\leq w\leq b\\ 0 & \mathrm{otherwise}. \end{array}\right.$$

We can decompose the projector *P*(*a*, *b*) in the following manner. First, we define a projector for functions *ϕ* in H with coordinates *ϕ*(*w*) defined on the real-valued interval [*l*, *a*], *l* < *a* as

$$M(a)\cdot \phi =\phi_{a}$$

and

$$\phi_{a}(w)=\left\{ \begin{array}{ll} \phi (w) & l\leq w\leq a\\ 0 & \mathrm{otherwise}. \end{array}\right.$$

Then the tensor product forms a projector in $H^{2}$ defined by *M*(*a*)⊗*M*(*b*) = *P*(*a*, *b*). The projector for empirical events produced by the pair of sensors *X*, *Z* on a rectangle [*l*, *x*] × [*l*, *z*] is then constructed from a tensor product of unitary operators

$$\begin{array}{rl} P_{X\cap Z}(x,z) & =(U_{X}⊗U_{Z})(M(x)⊗M(z)){\left(U_{X}⊗U_{Z}\right)}^{†}.\\ & =(U_{X}M(x)U_{X}^{†})⊗(U_{Z}M(z)U_{Z}^{†})\\ & =(R_{X}(x)⊗I)\cdot (I⊗R_{Z}(z))\\ & =P_{X}(x)\cdot P_{Z}(z). \end{array}$$

(Note *R*_*X*_(*x*) = *U*_*X*_ · *M*(*x*) · *U*^†^_*X*_ operates in H and *P*_*X*_(*x*) = *R*_*X*_(*x*)⊗*I* operates in $H^{2}$, and that the projectors *P*_*X*_(*x*) and *P*_*Z*_(*z*) in this section are the same as those used in §4c.)

Likewise, the projector for events produced by the pair of sensors *Y*, *Z* on the rectangle is then constructed from a tensor product of unitary operators

$$P_{Y\cap Z}(y,z)=(U_{Y}⊗U_{Z})(M(y)⊗M(z)){\left(U_{Y}⊗U_{Z}\right)}^{†}.$$

For the pair of sensors, *X*, *Y*, when *X* is measured first in the context [*x*, *y*], the projector is constructed from the tensor product

$$\begin{array}{rl} Q_{X,Y}(x,y) & =((U_{Y}M(y)U_{Y}^{†})⊗I)\cdot ((U_{X}M(x)U_{X}^{†})⊗I).\\ & =(U_{Y}M(y)U_{Y}^{†}U_{X}M(x)U_{X}^{†})⊗I. \end{array}$$

The measurement operator for observing sensor *X*, then sensor *Y*, and then sensor *Z* in context [*x*, *y*, *z*], for *X* and *Z* in the rectangle [*l*, *x*] × [*l*, *z*] and *Y* in the interval [*l*, *y*] is given by

$$Q_{X,Y,Z}(x,y,z)=((U_{Y}M(y)U_{Y})⊗I)\cdot P_{X\cap Z}(x,z).$$

One might ask about the case with two non-commuting pairs and one commutative pair of observables. This case does not occur if the basis changes across the three pairs. If *U*_*YX*_ is the unitary operator (other than identity) that transforms from the **X** basis to the **Y** basis (making **X** and **Y** non-commutative), and *U*_*ZY*_ is the unitary operator (other than identity) that transforms from the **Y** basis to the **Z** basis (making **Y** and **Z** non-commutative), and *U*_*ZY*_≠*U*^†^_*YX*_, then *U*_*ZY*_ · *U*_*YX*_≠*I* is the unitary operator that transforms from the **X** basis to the **Z** basis (making **X** and **Z** non-commutative). This case can occur if *U*_*ZY*_ = *U*^†^_*YX*_ so that *U*_*XZ*_ = *U*_*ZY*_ · *U*_*YX*_ = *I* making the observables **X** and **Z** share the same basis. In the latter case, the spectral values of one observable are a function of the spectral values of another observable. This occurs in physics, for example, when measuring position, momentum. and energy of a particle along the same direction in free space (assuming no potential function of position), in which case position does not commute with momentum or energy, but momentum commutes with energy. In this case, one can use the quantum rules for two of the non-commuting observables, say **X** and **Y**, and then apply standard formulae for the distribution of the transformation of variables to compute the distribution for the third variable **Z**.

## 6. Step 3: Constructing unitary transformations

The next problem concerns the construction of the unitary operator for space H. This is all we need because the unitary operator for space $H^{2}$ is obtained by a tensor product of unitary operators in space H.

A unitary operator can be constructed from a bounded self-adjoint operator *H* = *H*^†^ by the transformation

U=exp⁡(i⋅H).

The unitary operator is not self-adjoint, but it can be constructed from a self-adjoint operator. Therefore, we only need to determine a self-adjoint (Hermitian) operator *H*.

### (a) Kernel method

One method is to work with real compact Hermitian operators *H* such that

$$\int_{l}^{u}\int_{l}^{u}|H(x,y){|}^{2}\;dx\;dy\leq +\infty .$$

Then according to the spectral theorem, *H* can be decomposed in terms of its real eigenvalues {*λ*_*j*_, *j* = 1, …, ∞} that decrease toward zero in magnitude and its corresponding eigenvectors {*V*_*j*_, *j* = 1, …, ∞}, where each eigenvector has coordinate *V*_*i*_(*x*) at index value *x*. Each eigenvector corresponds to a one-dimensional projector *P*_*j*_ containing elements *P*_*j*_(*x*, *y*) = *V*_*j*_(*x*)V*_j_(*y*) and satisfying the properties *P*_*j*_ · *P*_*j*_ = *P*_*j*_ = *P*^†^_*j*_. Therefore, the unitary operator can be determined as follows:

$$\begin{array}{rl} H & =\sum \lambda_{j}P_{j},\\ H(x,y) & =\sum \lambda_{j}V_{j}(x)V_{j}^{*}(y),\\ U & =\sum e^{i\lambda_{j}}P_{j}\\ \mathrm{and}\;\;U(x,y) & =\sum e^{i\lambda_{j}}V_{j}(x)V_{j}^{*}(y). \end{array}$$

One method to form the Hermitian operator *H* is to use a kernel operator, which produces a real-valued, symmetric, positive semi-definite operator. For example, *H*(*x*, *y*) = e^−((*x*−*y*)/*σ*)^2^^ is a kernel with parameter *σ*. Methods for building kernels, using a small number of parameters, have been discussed extensively in the support vector machine literature [12] and in the literature on Gaussian processes [13]. Methods for numerical approximation of the eigenfunctions are given in [14]. We have not yet used the Kernel method; however, this method has been used successfully in numerous applications in cognitive science and machine learning.

### (b) Schrödinger equation method

Another method for constructing the unitary operator is to derive it from the Schrödinger equation. According to the Schrödinger equation (with a Hamiltonian that is constant across time), ***ψ***(*t*) = *U*(*t*) · ***ψ***(0), where

$$\begin{array}{rl} & \frac{d}{dt}\psi (t)=-i\cdot H\cdot \psi (t),\\ & \frac{d}{dt}U(t)\psi (0)=-i\cdot H\cdot U(t)\psi (0)\\ \mathrm{and}\;\; & \frac{d}{dt}U(t)=-i\cdot H\cdot U(t) \end{array}$$

and U(t)=exp⁡(−i⋅t⋅H) is the solution to the last equation above. This unitary operator transforms the state as a function of time, starting with U(0)=exp⁡(−i⋅0⋅H)=I. The unitary operator *U*(*t*) is essentially the same as the unitary operator used with the kernel method, except that we have changed the sign on the exponent that transforms the Hermitian operator into a unitary operator. The Hermitian operator *H* is called the Hamiltonian. Many different types of Hamiltonians are used in quantum physics, but the most common is the sum of kinetic and potential energy operators

$$H=-\sigma \cdot \frac{\partial^{2}}{\partial x}+V$$

and

$$H\cdot \psi (x)=-\sigma \cdot \frac{\partial^{2}}{\partial x}\psi (x)+V(x)\cdot \psi (x),$$

where *V* is the potential operator and *V* (*x*) is the potential function. For example, a quadratic potential for the harmonic oscillator is defined by *V* (*x*) = *x*^2^. Boundary conditions must also be defined to determine a solution. Methods for deriving the eigenvalues and eigenfunctions for Hamiltonians are described in standard textbooks on quantum theory [11]. However, in many cases, analytic solutions cannot be obtained, and instead numerical methods for solving partial differential equations are used. As described in §8, we have successfully used this method to model context effects obtained with approximately continuous measurements of human judgements.

## 7. Step 4: Constructing an initial state

The state of a quantum probability model is defined by a projector *ρ*, which is the projector corresponding to a ray spanned by the vector ***ψ***. (However, see footnote 1.) We still need a method to determine this state. Generally, this depends on prior knowledge of the system, which depends on a specific application. In psychological applications, this represents the prior state of beliefs of a person, analogous to *a prior* probability distribution in a Bayesian cognition model.

One simple type of initial state is a real-valued, uniform amplitude distribution on a small bounded interval inside [*l*, *u*]. Another useful type of initial state is one that is constructed from a complex-valued Gaussian function. Suppose *ψ*(*x*) is defined by a truncated Gaussian distribution with

7.1 $$\psi (x)=C\cdot e^{i\cdot p\cdot x}\cdot e^{-(1/2){\left((x-\mu )/\sigma \right)}^{2}},$$

inside the interval [*l*, *u*] and zero outside. The constant *C* is used to normalize the squared magnitudes of the truncated Gaussian. The initial probability distribution, generated by the squared magnitudes of the amplitudes, is (approximately because of truncation) a real Gaussian distribution. The parameter *μ* determines the mean of this initial probability distribution, and the parameter $\sigma /\sqrt{2}$ determines the standard deviation. These two parameters can be chosen in such a way that almost all of the distribution within the truncated Gaussian (before normalization) is contained in the interval [*l*, *u*]. The parameter *p* determines the mean momentum of the quantum process when there is no potential function, *V* = 0. If *p* > 0, then the Gaussian distribution moves across time to the right at a rate equal to *p* and the variance increases proportional to *t*^2^. If *p* = 0, then there is no momentum.

If *ψ*(*x*, *y*) is defined on a rectangle within *R*^2^, then we can construct the initial state from the tensor product ***ψ***_*X*_⊗***ψ***_*Y*_ with coordinate *ψ*(*x*, *y*) = *ψ*_*X*_(*x*) · *ψ*_*Y*_(*y*) at index (*x*, *y*), and with *ψ*_*X*_(*x*) and *ψ*_*Y*_(*y*) determined by equation (7.1) using different parameters.

One justification for using a uniform initial state or a Gaussian initial state is that analogous assumptions have been made about the initial state of Markov processes, which are commonly used in cognitive science. In our applications of HSM theory to continuous measures, we compare the HSM model with Markov models, and we try to use parallel assumptions. More details about this are described in the next section.

## 8. Empirical applications

Recently, we reported two applications of the principles described above. Both applications concern the topic of belief change while monitoring evidence. The ‘classical’ approach in cognitive science to this problem is to assume that the dynamics follow a Markov process, in particular a drift diffusion process. A ‘non-classical’ approach that we have implemented is to assume that the dynamics follow a quantum process, in particular, a Schrödinger type process.

Previously, we [15] empirically compared the predictions of these two models using a ‘dot motion’ task for studying evidence monitoring. This is a perceptual task that requires participants to judge the left/right direction of dot motion in a display consisting of moving dots within a circular aperture.

The experimental design used two different contexts of judgement. In the choice-confidence condition, participants were given *t*_1_ = 0.50 s to view the display, and then a tone was presented that signalled the time to make a binary (left/right) decision. After an additional Δ*t* = 0.50, 0.75, or 1.50 s following the choice, participants were prompted by a second tone to make a confidence rating on an approximately continuous 0 (certain left) to 100% (certain right) rating scale. In a confidence-only condition, participants did not have to make any decision. Instead, they simply made a pre-determined response when hearing the tone at time *t*_1_, and then later they made a confidence rating at the same total time points *t*_2_ = *t*_1_ + Δ*t* as the choice-confidence condition.

According to a Markov model, the marginal distribution of confidence at time *t*_2_ (pooled across choices at time *t*_1_ for the choice-confidence condition) should be the same between the two conditions at time *t*_2_. According to the quantum model, the decision at time *t*_1_ produces a collapse, which introduces interference during processing before the second judgement, and this interference disturbs and changes the marginal distribution of confidence for the choice-confidence condition at time *t*_2_. The results strongly favoured the quantum model predictions: the interference effect (i.e. violation of marginal invariance at time *t*_2_ across contexts for the confidence ratings) was significant at the group level, and a majority of participants produced significant interference effects at the individual level as well.

More to the point of this presentation, the Markov and quantum models were used to quantitatively predict the continuously distributed confidence ratings. The Markov process was described by a Kolmogorov forward equation. Define *p*(*t*, *x*) as the probability density for confidence level *x* at time *t*.

$$\frac{\partial }{\partial t}p(t,x)=\frac{\sigma^{2}}{2}\cdot \frac{\partial^{2}}{\partial x^{2}}p(t,x)-\mu \cdot \frac{\partial }{\partial x}p(t,x).$$

The process was parameterized by constant mean drift *μ* and diffusion *σ* parameters. The quantum process was described by a Schrödinger equation. Define *ψ*(*t*, *x*) as the amplitude density for confidence level *x* at time *t*.

$$i\frac{\partial }{\partial t}\psi (t,x)=-\sigma^{2}\frac{\partial^{2}}{\partial x^{2}}\psi (t,x)+\mu \cdot x\cdot \psi (t,x),$$

also with a diffusion parameter *σ* and a linear potential function with slope parameter *μ*. Both models used a uniform initial distribution. Both models employed the same number of parameters. The results of a Bayesian model comparison strongly favoured the quantum over the Markov model for a majority of participants.^2^

In a more recent experiment, [17] we examined the sequential effects of one continuous confidence judgement on a later judgement. Once again, the participants monitored dot motion. In this new experiment, two confidence ratings were made at a pair (*t*_1_, *t*_2_) of time points. The experiment included three main conditions: (1) requests for confidence ratings at times *t*_1_ = 0.50 s and *t*_2_ = 1.50 s, (2) requests for ratings at times *t*_1_ = 1.50 s and *t*_2_ = 2.50 s, and (3) requests for ratings at times *t*_1_ = 0.50 s and *t*_2_ = 2.50 s.

First of all, this design provided another test for interference effects by comparing the marginal distribution of probability ratings at time *t*_2_ = 1.50 s for condition 1 (pooled across ratings made at time *t*_1_ = 0.50 s) with the distribution of ratings at time *t*_1_ = 1.50 s from condition 2. Once again, the Markov model predicts no difference between conditions at the matching time points, whereas the quantum model predicts an interference effect of the first rating on the second. Only a minority of participants produced significant interference effects in this study. Apparently, the act of making a resolute decision produces greater interference than an irresolute confidence judgement at the first measurement.

This design also provided a generalization test for quantitatively comparing the predictions computed from the competing models. The parameters from both models were estimated from the confidence ratings distributions obtained from the first two conditions for each individual; then these same parameters were used to predict the confidence rating distribution for each person on the third condition.

The Markov model was again based on the Kolmogorov forward equation, and the quantum model was again based on the Schrödinger equation. Both models used a Gaussian initial distribution in this new comparison (*p* = 0). Both models used two parameters to predict the confidence rating distributions: *μ*, *σ*. We estimated these two parameters from the joint distribution (pair of ratings at 0.5 s and 1.5 s) obtained from condition 1, and the joint distribution (pair of ratings at 1.5 s and 2.5 s) from condition 2, separately for each participant. Then we used these same two parameters to predict the joint distribution (pair of ratings 0.5 s and 2.5 s) obtained from condition 3 for each participant. The results of the new experiment indicated that the ratings of the majority of participants were better predicted by the quantum model than the Markov model. Together, these results indicate that the principles described in §§4–7 are viable for predicting continuous human judgements.

## 9. Conclusion

The problem we addressed was to build an HSM model, based on quantum probability theory, for a collection of probability distributions obtained from subsets of variables. Past applications were developed for discrete variables with finite-dimensional Hilbert spaces, and the present work extends the theory to continuous variables with infinite-dimensional spaces. Here, we used an example involving subsets of three continuous variables, *X*, *Y*, *Z*.

The application of a quantum model requires the following steps (see [18] and [19]). First, the compatibility between pairs of variables needs to be established, and one way to do this is to test for order effects. Second, a projector for each variable needs to be built. The projector is built from a unitary operator, and the unitary operator is built from a Hermitian operator. Finally, an initial state must be estimated.

The Hermitian operator can be constructed using either a kernel method similar to those used in machine learning or by solving a Schrödinger equation similar to that used in quantum mechanics. Either method will involve some unknown model parameters that need to be estimated from the data.

The initial state can be constructed using a complex-valued Gaussian function. Again, this will involve some unknown parameters that need to be estimated from the data.

The parameters used to define the Hermitian operators and the initial state can be estimated from the data using maximum-likelihood or Bayesian estimation methods. We briefly reviewed several applications of these methods to continuous variables. We hope the extension to continuous variables will initiate new empirical work with continuous variables.

## Footnotes

1 More generally, a state is a positive operator with trace equal to one. A convex combination of states is also a state.

2 Both models were implemented using an approximation method to solve the partial differential equations described in the appendix of [16].

## Data accessibility

This article has no additional data.
